# The BET inhibitor JQ1 selectively impairs tumour response to hypoxia and downregulates CA9 and angiogenesis in triple negative breast cancer

**DOI:** 10.1038/onc.2016.184

**Published:** 2016-06-13

**Authors:** L L da Motta, I Ledaki, K Purshouse, S Haider, M A De Bastiani, D Baban, M Morotti, G Steers, S Wigfield, E Bridges, J-L Li, S Knapp, D Ebner, F Klamt, A L Harris, A McIntyre

**Affiliations:** 1 Molecular Oncology Laboratories, Weatherall Institute of Molecular Medicine, University of Oxford, Oxford, UK; 2 Department of Biochemistry/UFRGS, Porto Alegre, Brazil; 3 CAPES Foundation, Ministry of Education of Brazil, Brasilia, Brazil; 4 High Throughput Genomics, Wellcome Trust Centre for Human Genetics, University of Oxford, Oxford, UK; 5 Institute of Translational and Stratified Medicine, Plymouth University, Peninsula Schools of Medicine and Dentistry, Plymouth, UK; 6 Nuffield Department of Clinical Medicine, Structural Genomics Consortium, University of Oxford, Oxford, UK; 7 Goethe University Frankfurt, Institute for Pharmaceutical Chemistry and Buchmann Institute for Life Sciences, Campus Riedberg, Frankfurt, Germany; 8 Nuffield Department of Medicine, Target Discovery Institute (TDI), University of Oxford, Oxford, UK; 9 Cancer Biology, Division of Cancer and Stem Cells, The University of Nottingham, Nottingham, UK

## Abstract

The availability of bromodomain and extra-terminal inhibitors (BETi) has enabled translational epigenetic studies in cancer. BET proteins regulate transcription by selectively recognizing acetylated lysine residues on chromatin. BETi compete with this process leading to both downregulation and upregulation of gene expression. Hypoxia enables progression of triple negative breast cancer (TNBC), the most aggressive form of breast cancer, partly by driving metabolic adaptation, angiogenesis and metastasis through upregulation of hypoxia-regulated genes (for example, carbonic anhydrase 9 (*CA9*) and vascular endothelial growth factor A (*VEGF-A*). Responses to hypoxia can be mediated epigenetically, thus we investigated whether BETi JQ1 could impair the TNBC response induced by hypoxia and exert anti-tumour effects. JQ1 significantly modulated 44% of hypoxia-induced genes, of which two-thirds were downregulated including *CA9* and *VEGF-A*. JQ1 prevented HIF binding to the hypoxia response element in *CA9* promoter, but did not alter HIF expression or activity, suggesting some HIF targets are BET-dependent. JQ1 reduced TNBC growth *in vitro* and *in vivo* and inhibited xenograft vascularization. These findings identify that BETi dually targets angiogenesis and the hypoxic response, an effective combination at reducing tumour growth in preclinical studies.

## Introduction

Epigenetic regulators are promising targets in cancer as transcriptional dysregulation and mutations in chromatin modulators and transcription factors (TF) are common in many malignancies. The bromodomain and extra-terminal (BET) proteins are lysine acetylation readers that mediate gene expression, including oncogenes.^1^ BET inhibitors (BETi) demonstrate many anticancer effects by downregulating gene expression of oncogenic factors.^2^ Breast cancer is the most common female cancer and triple negative breast cancer (TNBC) is its most aggressive subtype. Low oxygen (hypoxia) can drive TNBC progression,^3^ promoting adaptation through genes within the major hallmarks of cancer.^4^ Hypoxia can control gene expression recruiting chromatin remodelling complex^5^ and histone deacetylases.^6^ Thus, we investigated whether BETi JQ1 could impair the hypoxia response in TNBC and exert therapeutic effects.

Hypoxia is found in >50% of breast tumours and arises from high metabolic and proliferative rates and aberrant tumour vascularization. Clinically, hypoxia is associated with chemo-radiotherapy resistance, metastasis and poor survival,^7^ being a key area for targeted therapeutic development.^3, 8^ Most hypoxic responses are mediated by the hypoxia-inducible factors 1α and 2α (HIF-1α and HIF-2α), which in the absence of O_2_ stabilize and heterodimerise with HIF-1β.^7, 9^ This heterodimer binds to the hypoxia response element in gene promoters and induces transcription of genes, which drive molecular adaptation through many pathways, including pH regulation (CA9), angiogenesis (VEGF-A), metabolism (LDHA) and metastasis (LOX).^7, 8^ Other pathways including the unfolded protein response, XBP1 and ATF4 are also important.^8, 10^ TNBC shows overexpression of HIF target genes and is the breast cancer subtype most frequently associated with hypoxia.^11, 12^ Targeting HIF directly is a major challenge, while targeting HIF downstream targets such as VEGF has proven more feasible, although targeting just one protein has had less effect on overall survival than expected.^13, 14, 15^

The BET proteins (BRD2-4 and BRDT) regulate transcription by ‘reading' acetylated histones and recruiting TFs and epigenetic regulators.^1, 2^ JQ1 is a BETi^16^ that showed effects on tumour growth and survival,^17^ cell cycle arrest, and differentiation.^16, 18, 19, 20^ Although many attributed JQ1 effect to its MYC downregulation,^18, 21^ it is unlikely that this is the sole mechanism^17^ and some studies do not corroborate this,^22^ as MYC downregulation is not always sufficient to inhibit cell growth^23^ and JQ1 effects are observable without MYC alteration.^24^ BET proteins can associate with many TFs^21, 25^ and other genes are regulated by BETi, such as p21, BCL-xl, BCL2, AKT, FOSL1 and RUNX2.^20, 21, 22, 24^ Although the oncogenic driver varies, tumour addiction to BET activation seems common to many malignancies. These results led to clinical evaluation and there are 13 BETi clinical trials currently underway (www.clinicaltrials.gov).

Given the clinical investigation of BETi, we assessed whether the BETi JQ1 could alter the hypoxia response, exerting an anti-tumour effect. JQ1 modulated 44% of hypoxia-responsive genes, of which two-thirds were downregulated including *CA9* and *VEGF-A*. JQ1 reduced TNBC growth in monolayer and spheroid culture. Furthermore, JQ1 prevented HIF binding to the *CA9* promoter. Finally, JQ1 downregulated *CA9* and *VEGF-A* expression and reduced growth and vascularization (CD31 positivity) in a TNBC xenograft model. These data show that JQ1 impairs tumour response to hypoxia.

## Results

### JQ1 downregulates the hypoxic transcriptome response

**Figure 1:**
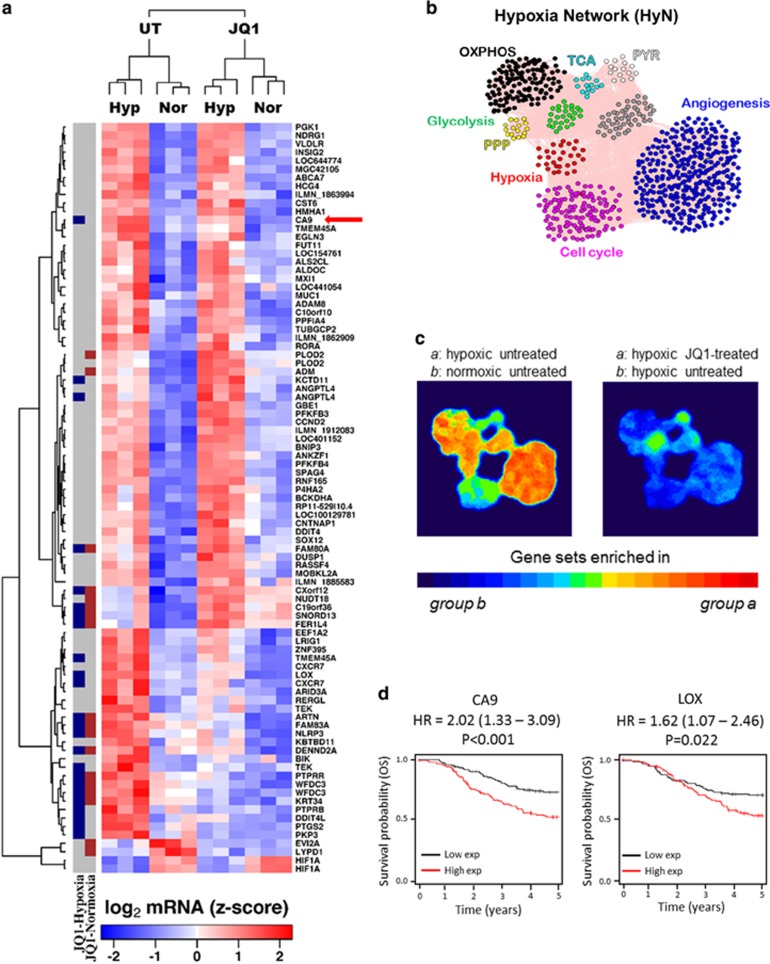
JQ1 downregulates the expression of several hypoxia-regulated genes, especially CA9. (**a**) List of DEG under hypoxia in MDA-MB-231 cells obtained from microarray. Columns at the left denote DEG under JQ1 treatment, either in normoxia (red blocks) or hypoxia (blue dots). CA9 is the most prominently downregulated gene in hypoxia (red arrow). (**b**) Hypoxia Network (HyN) created including pathways regulated by hypoxia. Grey dots are linking genes, included to the network remains stable. (**c**) Most components of HyN are upregulated by hypoxia and downregulated by JQ1 treatment in MDA-MB-231 cells. (**d**) Kaplan–Meier curves demonstrating the prognostic value of two genes consistently inhibited by JQ1 in both cell lines tested (MDA-MB-231 and MCF-7) for triple negative breast cancer patients. OXPHOS, oxidative phosphorylation; TCA, tricarboxylic acid cycle; PYR, pyruvate metabolism; PPP, pentose phosphate pathway.

We performed gene array analysis after JQ1 treatment in hypoxia or normoxia in the TNBC cell line MDA-MB-231 (Figure 1) and the ER+ MCF-7, as the hypoxic transcriptome of this cell line is well-documented^26^ (Supplementary Figure S1). JQ1 alone profoundly affected gene expression (fold change (FC) log2⩾1 and *P*<0.05, *n*=3): 2338 genes were differentially expressed (DEG) in MDA-MB-231 and 2993 in MCF-7; while hypoxia induced 119 DEG in MDA-MB-231 and 1286 in MCF-7. Within the DEG in hypoxia, a considerable proportion were modulated by JQ1 in hypoxia, 44% in MDA-MB-231 and 29% in MCF-7 (Figure 1a and Supplementary Figure S1A). Interestingly, JQ1 had a greater impact on hypoxia-sensitive genes than those that were not hypoxia-sensitive (*P*=0.046; median logFC=1.22 × 1.02) (Supplementary Figure S1C).

To further investigate the effect of JQ1 on tumour response to hypoxia, we defined which pathways were hypoxia-regulated and evaluated their expression under JQ1 treatment. Then, we developed a Hypoxia Network (HyN) (Figure 1b) containing the Hypoxia Signature^27^ and a list of genes for each pathway found to be hypoxia-regulated (obtained from KEGG, Supplementary Table S1). Hypoxia upregulated most of the HyN clusters in both cell lines (Figure 1c and Supplementary Figure S1B). Gene Set Enrichment Analysis showed that hypoxia upregulates angiogenesis, glycolysis, oxidative phosphorylation and pentose phosphate pathway in MDA-MB-231 (Figure 1c and Supplementary Table S2). The Hypoxia Signature^27^ set of genes was upregulated in hypoxia as expected (MDA-MB-231: ES=−0.79, normalized enrichment score=−2.68, *P*<0.001, false discovery rate q-value<0.001; MCF-7: ES=−0.83, normalized enrichment score=−2.63, *P*<0.001, false discovery rate q-value<0.001, *n*=3). JQ1 treatment prevented hypoxic upregulation of the hypoxia signature, angiogenesis, oxidative phosphorylation and pentose phosphate pathway gene data sets, but did not alter glycolysis or MYC expression. In addition, JQ1 treatment downregulated the cell cycle and TCA sets of genes in hypoxia (Figure 1c and Supplementary Table S2).

MCF-7 results confirmed most of these findings (Supplementary Figure S1B and Supplementary Table S3). CA9 was the most significantly downregulated gene in both cell lines (MDA-MB-231: logFC=−1.40, *P*=2.3 × 10^−3^; MCF-7: logFC=−4.22, *P*=1.51 × 10^−9^, *n*=3) (Figure 1a). The prognostic value in TNBC of the consistently DEG by JQ1 in hypoxia in both tested cell lines was evaluated (Figure 1d and Supplementary Figure S2) on analysis of TCGA data sets http://cancergenome.nih.gov/. Two of them are associated with poor prognosis in TNBC: CA9 (hazard ratio=2.02; *P*=0.001) and LOX (hazard ratio=1.62; *P*=0.022) (Figure 1d).

### JQ1 reduces TNBC monolayer and spheroid growth

**Figure 2:**
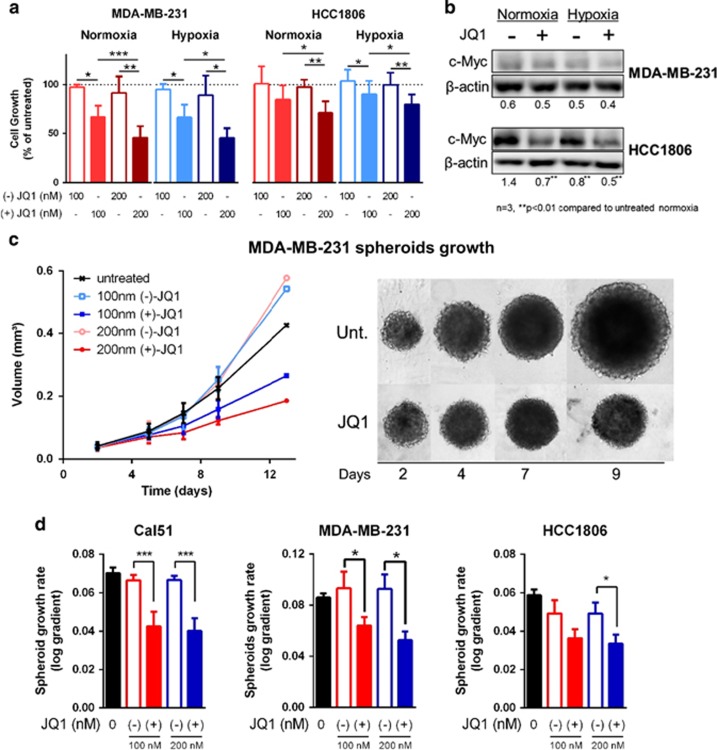
JQ1 reduces TNBC monolayer and spheroid growth, regardless of MYC. (**a**) Cell cultures were grown in 96-well plates for 72 h in each condition. (+)-JQ1 dose-dependently reduces monolayer (two-dimensional) cell growth of TNBC cell lines MDA-MB-231 and HCC1806 in normoxia and hypoxia, while (−)-JQ1 does not. (**b**) JQ1 reduced c-Myc immunocontent only in the MYC-amplified cell line HCC1806, while the non-mutated cell line MDA-MB-231 showed no difference in c-Myc. (**c**) Representative spheroid growth curve and pictures of MDA-MB-231 spheroids following JQ1 treatment. (**d**) (+)-JQ1 reduces spheroid growth in MDA-MB-231 and other TNBC cell lines, while (−)-JQ1 does not. One-way analysis of variance, *n*=3, *P<0.05, **P<0.01, ***P<0.001.

JQ1 dose-dependently reduced cell growth in monolayer cultures in all the four TNBC cell lines tested, in hypoxia and normoxia (Figure 2a and Supplementary Figure S3A). The non-active enantiomer (−)-JQ1^16^ showed no effect on cell growth (Figure 2a and Supplementary Figure S3A).

MYC amplification was not a predictor of JQ1 sensitivity (Figure 2b). JQ1 reduced growth and c-Myc expression of HCC1806 (*P*<0.01, *n*=3), which has MYC amplification^28^ and the highest MYC expression among the investigated cell lines. However, JQ1 did not alter c-Myc, yet induced a similar growth inhibition in MDA-MB-231 (Figure 2b and Supplementary Figure S3B), which has no MYC amplification (cBioPortal).^28, 29^ JQ1 induced a similar growth inhibition in two additional MYC-non-amplified TNBC cell lines: Cal51 and SUM159 (Supplementary Figure S3A). No differential effect of JQ1 on cell growth in two-dimensional culture in normoxia or hypoxia was identified.

JQ1 inhibited growth of tumour spheroids in all the cell lines (Figures 2c and d and Supplementary Figure S3C). This model is more physiologically relevant, as it creates the oxygen, nutrient and pH gradients found in tumours, and improves the translation of drug candidates.^30^ SUM159 did not grow as spheroids. JQ1 reduced spheroid growth rate in MDA-MB-231 (*P*<0.05, *n*=3), HCC1806 (*P*<0.05, *n*=3) and Cal51 (*P*<0.01, *n*=3). Once again, the non-active enantiomer (−)-JQ1 did not cause any significant effect (Figures 2c and d and Supplementary Figure S3C).

### JQ1 reduces CA9 and Ki67 expression in TNBC spheroids

**Figure 3:**
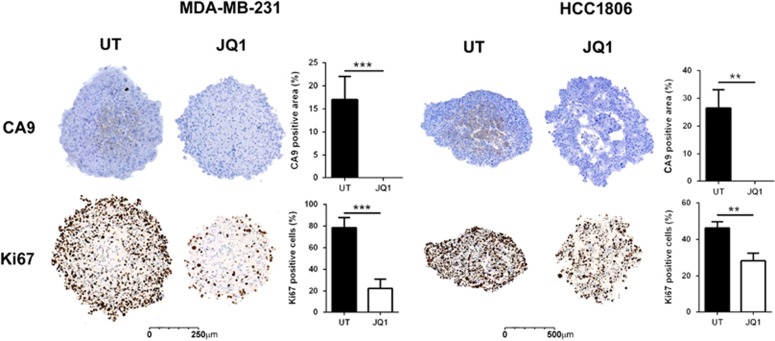
JQ1 reduces CA9 and Ki67 stain in TNBC spheroids. CA9 expression is visible in untreated (UT) spheroids, but undetectable in JQ1-treated spheroids. The proliferative marker Ki67 was also reduced in spheroids treated with JQ1. For immunohistochemistry, spheroids were fixed with formalin 10%, embedded in agarose, processed, embedded in wax and cut with a microtome. Student *t*-test, *n*=3, **P<0.01, ***P<0.001.

Immunohistochemistry showed that untreated spheroids express CA9 in their hypoxic cores, while JQ1-treated spheroids did not (MDA-MB-231: *P*<0.001, *n*=3; HCC1806: *P*<0.01, *n*=3) (Figure 3). JQ1-treated spheroids had lower Ki67 staining, indicating an anti-proliferative effect (MDA-MB-231: *P*<0.001, *n*=3; HCC1806: *P*<0.01, *n*=3) (Figure 3). This is concordant with the downregulation of cell cycle genes by JQ1 in the expression array analyses (Figure 1c, Supplementary Figure S1B and Supplementary Tables S2 and S3).

### JQ1 reduces expression of CA9, VEGF-A and additional hypoxia upregulated genes

**Figure 4:**
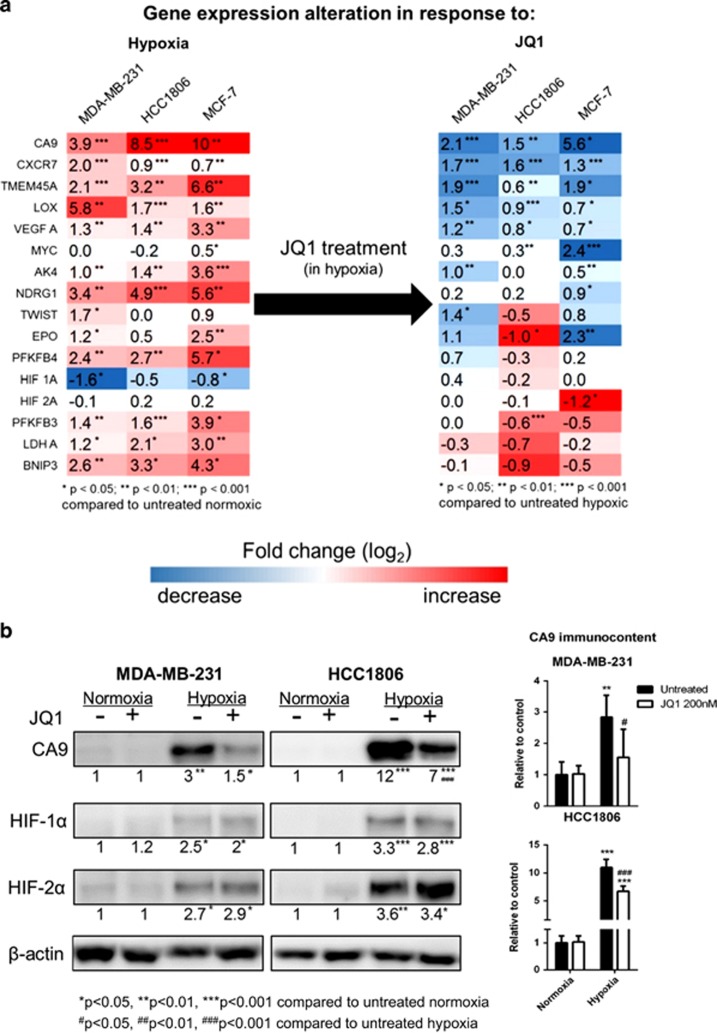
JQ1 reduces CA9 expression in TNBC cell lines. (**a**) Hypoxia upregulates several genes and JQ1 downregulates a group of them, CA9 being the most prominent across the three cell lines. HIF expression is not altered by JQ1. (**b**) CA9 is consistently downregulated by JQ1 in hypoxia, without any effect on HIF. Cells were treated with JQ1 for 24 h prior to RNA or protein extraction, and then gene expression was assessed by RT–qPCR and protein immunocontent was assessed by western blot. Two-way analysis of variance, *n*=3, *P<0.05, **P<0.01, ***P<0.001.

Quantitative PCR (qPCR) confirmed the JQ1-induced downregulation of hypoxia-responsive genes. *CA9* expression was consistently inhibited by JQ1, without alteration of HIF expression (mRNA and protein) in all cell lines tested (Figure 4 and Supplementary Figure S4A).

A panel of 16 genes was investigated including CA9, HIF-1α, HIF-2α, MYC and genes differentially expressed in the array analysis (Figure 1). All the HIF targets included in this panel are upregulated in hypoxia (Figure 4a) and many of them are downregulated by JQ1 treatment (Figure 4b). Within this panel, five genes were consistently downregulated by JQ1 treatment in hypoxia in all cell lines investigated, of which CA9 was the most prominent (MDA-MB-231: logFC=−2.1, *P*<0.001; HCC1806: logFC=−1.5, *P*<0.01; MCF-7: logFC=−5.6, *P*<0.05). The other four genes consistently downregulated are VEGF-A, CXCR7, TMEM45A and LOX. (Figure 4b). Other HIF-regulated genes such as LDHA or BNIP3 were not affected by JQ1 (Figure 4a), indicating a specific effect on a subset of the hypoxia transcriptome. MYC mRNA was downregulated in response to JQ1 treatment in normoxia and hypoxia, but only in MYC-amplified cell lines HCC1806 and MCF-7 (Figure 2b, Supplementary Figure S3B, Figure 4a and Supplementary Figure S4).

Additional BETi were tested (I-BET151 and I-BET762) and confirmed these findings, also downregulating CA9, VEGF, CXCR7, TMEM45A and LOX in hypoxia (Supplementary Figure S5). In contrast, I-BET151 in hypoxia induced VEGF-A in HCC1806 cell line and PFKFB3 in both cell lines tested. No effect was observed on HIF-1α expression, but there was a significant upregulation of HIF-2α at the RNA level.

Immunoblot analysis further confirmed that JQ1 induced a significant reduction of CA9 protein induction in hypoxia in both TNBC cell lines (*P*<0.01, *n*=3) (Figure 4b). JQ1 did not significantly alter HIF-1α and HIF-2α protein expression (Figure 4b).

### JQ1 reduces HIF binding to the CA9 promoter

**Figure 5:**
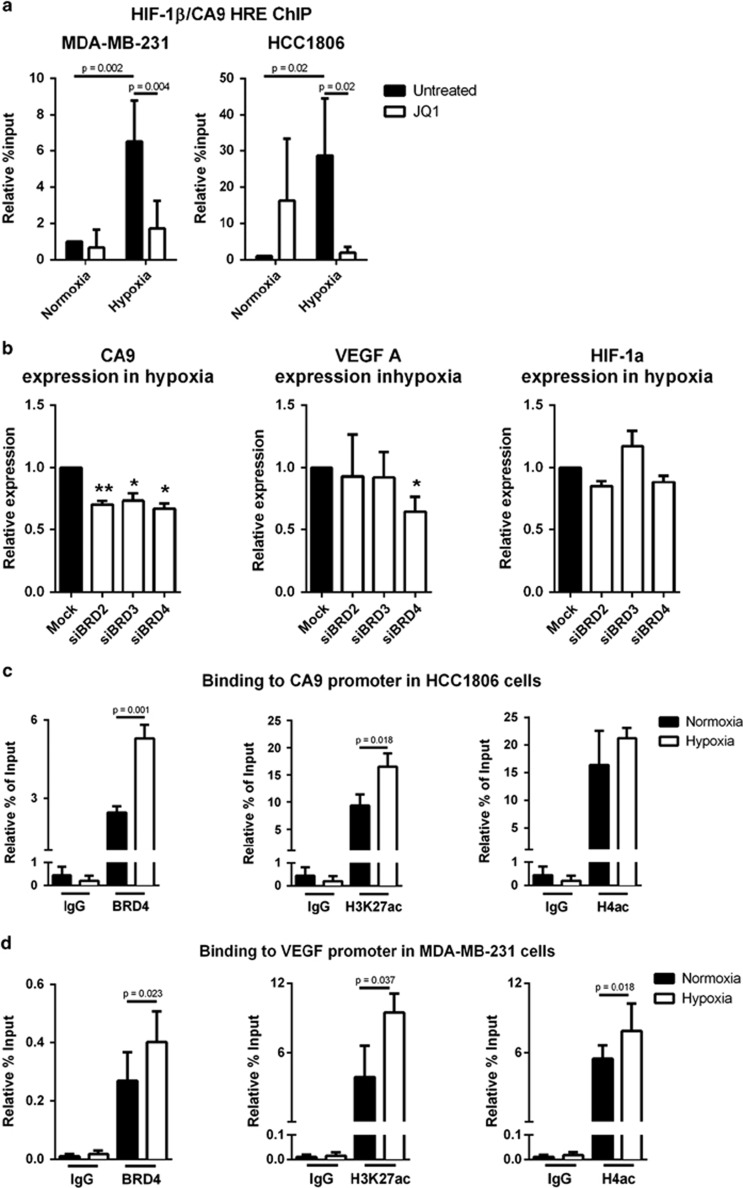
JQ1 prevented HIF binding to the CA9 promoter and BET protein knockdowns phenocopy JQ1 treatment. (**a**) ChIP assay for CA9 promoter region with HIF-1β immunoprecipitation in two TNBC cell lines. Two-way analysis of variance, *n*=3. (**b**) Expression of CA9, VEGF-A and HIF-1α in hypoxia following siRNA knockdown of BET proteins. One-way analysis of variance, *n*=3. (**c**) ChIP assay for CA9 promoter region with BRD4, H3K27 acetylation or H4 acetylation immunoprecipitation in HCC1806. *T*-test, *n*=3. (**d**) ChIP assay for VEGF-A promoter region with BRD4, H3K27 acetylation or H4 acetylation immunoprecipitation in MDA-MB-231. *T*-test, *n*=3 (H3K27 acetylation) *n*=5 (BRD4 and H4 acetylation), *P<0.05; **P<0.01.

To investigate how JQ1 prevents hypoxia-responsive gene expression, we evaluated chromatin immunoprecipitation (ChIP) of HIF-1β to the hypoxia response element of the CA9 promoter, as HIF-1α dimerizes with HIF-1β prior to transcription induction. As expected, hypoxia increased HIF binding in both the MDA-MB-231 (*P*=0.002, *n*=3) and HCC1806 (*P*=0.02, *n*=3) (Figure 5a). JQ1 treatment in hypoxia reduced HIF binding to the *CA9* promoter to normoxic levels in both MDA-MB-231 (*P*<0.01, *n*=3) and HCC1806 (*P*<0.05, *n*=3) (Figure 5a). This suggests HIF-1 is BET-dependent for binding/recruitment in some of its downstream targets, explaining how BET inhibition reduces the expression of hypoxia-induced genes.

In addition to this, expressions of *BRD2–*4 are increased in hypoxia (Supplementary Figure S6A), suggesting a role for BET proteins in hypoxia. Although JQ1 is a potent inhibitor for all the BET proteins,^16^ some of its effects are attributed to a specific isoform. We performed siRNA knockdown to investigate which isoform was responsible for these effects (Supplementary Figure S6B). BRDT was excluded as its expression was below the limit of detection. The BRDT siRNA did not change *CA9*, *VEGF* or *HIF-1α* expression. CA9 expression in hypoxia was reduced after BRD2 (*P*<0.01, *n*=3), BRD3 (*P*<0.05, *n*=3) and BRD4 (*P*<0.05, *n*=3) knockdown in MDA-MB-231 cells, without affecting HIF-1α level (Figure 5b). Only BRD4 (*P*<0.05, *n*=3) reduced VEGF-A expression in hypoxia (Figure 5b). Therefore, some HIF targets were shown to be BET-dependent (CA9 and VEGF-A), while others, such as LDHA are BET-independent.

To better comprehend how JQ1 can have these effects, BRD4 binding and acetylation of H3K27 and H4 in hypoxia at the promoters of VEGF and CA9 in HCC1806 and MDA-MB-231 were investigated by ChIP qPCR (Figures 5c and d and Supplementary Figures S6C and D). A significant increase of BRD4 binding was identified at the CA9 promoter in hypoxia in HCC1806 cells (*P*<0.01, *n*=3) (Figure 5c) and at the VEGF promoter in hypoxia in MDA-MB-231 cells (*P*<0.05, *n*=5) (Figure 5d). A significant increase for acetylation of H3K27 was found in response to hypoxia at the CA9 (*P*<0.05, *n*=3) and VEGF (*P*<0.01, *n*=3) promoters of HCC1806 cells (Figure 5c and Supplementary Figure S6C) and at the VEGF promoter of MDA-MB-231 (*P*<0.05, *n*=3) (Figure 5d). There was increased H4 acetylation in response to hypoxia at the VEGF promoter of MDA-MB-231 (*P*<0.05, *n*=5) (Figure 5d).

We investigated available BRD4 ChIP-Seq data for MCF-7 in normoxia^31^ and compared these with those genes that were downregulated by JQ1 in hypoxia and found that: 61% had BRD4 binding by ChIP-Seq in normoxia (Supplementary Tables S5 and S6). We investigated the BRD4 binding to genes that were downregulated by JQ1 in hypoxia and also upregulated in response to hypoxia; 39% of these genes showed direct binding by BRD4 (Supplementary Tables S5 and S6). Finally, we investigated the overlap between BRD4 binding in normoxia in MCF-7^31^ and HIF-1α or HIF-2α binding in hypoxia^26^ in MCF-7 published data sets. These data confirmed BRD4 and HIF-1 or HIF-2 binding in 14% of the genes that we identified are increased in hypoxia and downregulated by JQ1.

### JQ1 reduces tumour growth, CA9 and VEGF-A expression, and vascularization in TNBC xenografts

**Figure 6:**
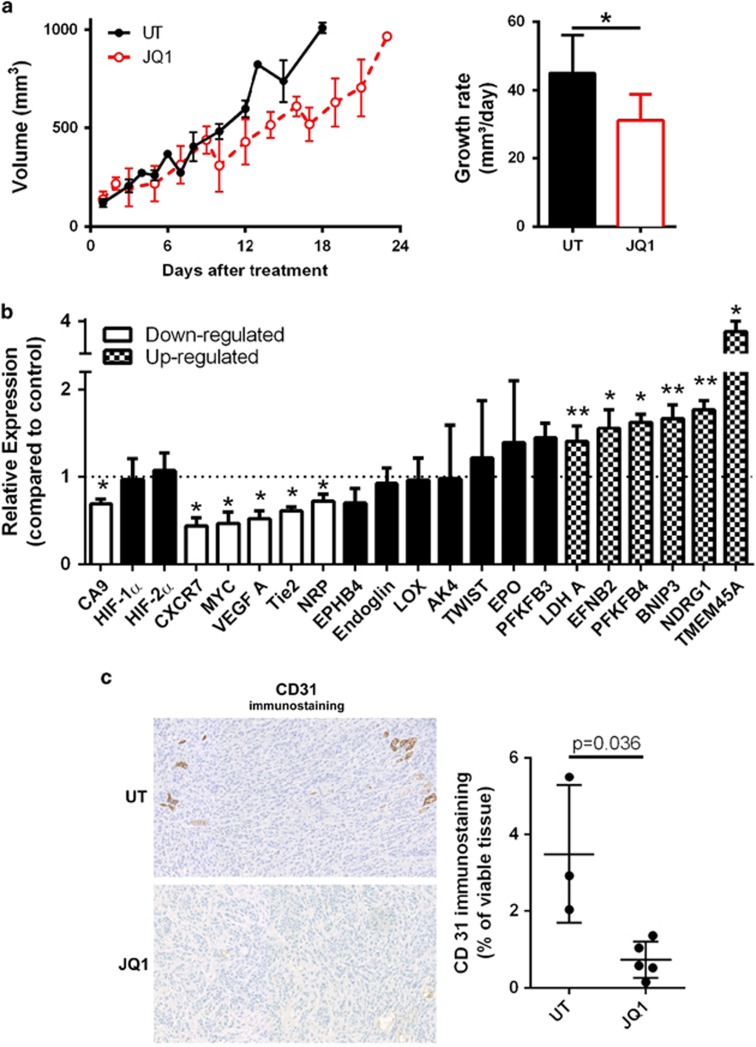
JQ1 reduces tumour growth, CA9, VEGF-A and additional angiogenesis-related gene expression and blood vessel count in xenograft model of TNBC. (**a**) Growth curve and rate in TNBC xenografts treated with JQ1 or untreated (UT). Linear regression followed by Student *t*-test, *n*=5, *P<0.05. (**b**) Gene expression in TNBC xenografts treated with JQ1, assessed by qRT–PCR analysis of RNA extracted from tumours. Student *t*-test compared with UT, *n*=3, *P<0.05, **P<0.01. (**c**) Representative CD31 immunostaining in TNBC xenografts. Non-parametric Mann–Whitney test, *n*=3 shCTL and *n*=5 JQ1-treated. Xenografts were grown using HCC1806 cells in 6–7-week-old female CD1 nude mice.

JQ1 (50 mg/kg) reduced HCC1806 xenograft growth (*P*<0.05, *n*=5) (Figure 6a) and expression of *CA9* (*P*<0.05, *n*=3), *VEGF-A* (*P*<0.05, *n*=3), *CXCR7* (*P*<0.05, *n*=3) and *MYC*, but did not affect the levels of *HIF-1α* or *HIF-2α* (Figure 6b).

Despite the downregulation effect observed in VEGF-A expression, angiogenesis is a broad phenomenon involving multiple molecular players.^7, 14, 15^ We thus investigated the expression of additional angiogenesis-related genes and observed JQ1-treated xenografts have lower expression of Tie2 (*P*<0.05, *n*=3) and NRP (*P*<0.05, *n*=3) and higher expression of EFNB2/ephrinB2 (*P*<0.05, *n*=3) (Figure 6b).

Interestingly, *LDH-A* (*P*<0.01, *n*=3), *BNIP3* (*P*<0.05, *n*=3), *PFKFB4* (*P*<0.01, *n*=3) and *TMEM45A* (*P*<0.05, *n*=3) (Figure 4a, Supplementary Figure S4 and Figure 6b) were unaltered or even downregulated by JQ1 (and other BETi) in cell culture and upregulated in xenografts.

We investigated the role of JQ1 in angiogenesis due to the consistent downregulation of *VEGF-A* found in cell lines (Figure 4a, Supplementary Figure S4) and the xenografts (Figure 6b) along with the reduction of the angiogenesis pathway expression (Figure 1c, Supplementary Figure S1B and Supplementary Tables S2 and S3). We observed that JQ1-treated xenografts had lower immunostaining of the blood vessel marker CD31 (*P*<0.05), thus indicating an anti-angiogenic effect of JQ1 (Figure 6c).

## Discussion

Hypoxia represents a key target for the development of therapies in cancer.^3, 32^ Hypoxia induces a transcriptomic shift largely dependent on HIF,^33^ and there is evidence for HIF dependence upon epigenetic regulation in response to hypoxia.^5, 6^ We demonstrate an epigenetic approach to modulate the tumour response to hypoxia and reduce growth in TNBC. JQ1 modulated the expression of 44% of hypoxia-responsive genes in MDA-MB-231 TNBC cell lines, of which two-thirds were downregulated. More specifically, JQ1 downregulated the expression of the major regulators of hypoxic pH regulation and angiogenesis, CA9 and VEGF-A, in TNBC cell lines and xenografts. We observed that in hypoxic conditions, there was an increased histone acetylation at, and BRD4 binding to, the CA9 and VEGF promoters, suggesting an explanation for JQ1 effectiveness in this context. It is possible that BRD2 and/or BRD3 may also be important in the regulation of *CA9* and *VEGF*. Analysis of published data sets^26, 31^ identified that many of the hypoxic JQ1-regulated genes have BRD4 binding and that 14% of the genes simultaneously upregulated by hypoxia, downregulated by JQ1 and bound by BRD4 are direct targets of either HIF-1α or HIF-2α these included *VEGFA* and *CA9*. Finally, as JQ1 prevents HIF binding to the *CA9* promoter but not all HIF-regulated transcription, the data suggest that some HIF targets are BET-dependent.

JQ1 consistently downregulated CA9 in *in vitro* and *in vivo* models. Hypoxic tumours develop in an acidic microenvironment, owing to increased production of metabolic acids and poor vascularization.^34^ CA9 is highly induced in hypoxia, where it allows adaptation to this environment maintaining a more neutral intracellular pH.^35, 36^ Increased CA9 expression is a marker of poor prognosis in breast cancer and is more common in TNBC than other breast cancer subtypes.^36^ CA9 inhibition reduces tumour growth and metastasis.^35, 36, 37^

JQ1 also consistently demonstrated an anti-angiogenic effect, as it reduced the expression of the angiogenic pathway, the key angiogenic inducer VEGF-A and blood vessel count. JQ1-treated xenografts showed lower levels of Tie2 and NRP, involved in vascular stabilization and branching and promotion of arterial growth.^38^ Conversely, there was a higher expression of EFNB2/ephrinB2 (Figure 6b), described as a regulator of arterial/venous specialization and vessel branching.^38^ Collectively, this indicates that JQ1 could impair the early steps of angiogenesis, a major hallmark of cancer.

Many studies showed an anti-tumoural effect of BETi,^19, 21, 22, 24^ and recently it was found that JQ1 shows a typical behaviour of anti-angiogenic agents and, in fact, JQ1 can reduce tumour vascularization by suppressing VEGF stimulation.^39^ Angiogenesis is upregulated by hypoxia and supports tumour progression.^7^ Anti-angiogenic therapy is a major cancer treatment strategy used to treat eight solid tumour types. However, this strategy was found to induce hypoxia in around 50% of patients.^7^ Hypoxic adaptation enables resistance to anti-angiogenic therapy and may in part explain why the promise of anti-angiogenic therapy in breast cancer has not been fulfilled. We have shown that combined inhibition of VEGF and CA9 act at least additively and in some examples synergistically to reduce tumour growth.^35^

Anti-angiogenic therapies can lead to metabolic adaptation.^7, 35^ JQ1 treatment increased the expression of LDHA and PFKFB4 in xenografts, but not in cell cultures. LDHA was reported to be downregulated by JQ1 in ovarian cancer,^40^ may be because of its regulation by MYC.^41^ Whereas JQ1 reduced the expression of oxidative phosphorylation, pentose phosphate pathway, TCA gene data sets but not glycolysis in TNBC cell line MDA-MB-231, in the ER+ cell line MCF-7, JQ1 increased TCA. This may be due to differences in the metabolic requirements of these subtypes of breast cancer. Thus, we might expect that a co-treatment with an anti-glycolytic or pro-OXSPHOS drug (such as metformin) could lead to a synergistic effect and be a promising therapy, especially in TNBC.

We also highlight the impact of BET inhibition on wider hypoxic gene expression. The hypoxic regions of tumours are resistant to other therapies, therefore we propose that utilizing BET inhibitors to target the hypoxic tumour cells in combination with additional chemotherapy or radiotherapy may provide better responses. Combining hypoxia targeting with radiotherapy or chemotherapy has been shown previously to provide a greater therapeutic response. For example, targeting hypoxia-regulated genes including CA9, one of the JQ1-regulated genes, increases sensitivity to radiotherapy and chemotherapy.^37, 42^

Stem cell characteristics comprise another important hallmark of cancer and epigenetic regulation has an important role in this. BRD4 has been proposed as a marker for self-renewal^43^ and JQ1 can downregulate genes involved in this process in human cord-derived mesenchymal stem cells.^44^ Stem cells are maintained in an undifferentiated state through expression of the core transcriptional factors Nanog, Oct4 and Sox2. BRD4 is required for Nanog expression and JQ1 inhibits this inducing rapid differentiation of murine embryonic stem cells^45, 46^ as well as significantly downregulating Oct4 and SOX2.^46^ BET inhibition or BRD4 depletion reduces the expression of pluripotent genes and shifts cellular fate.^45, 46^ Collectively, these data show that BRD4 is critical for the maintenance of pluripotency and maintaining stem cell fate, while inhibition of BET proteins enhances differentiation.

Initially, studies described JQ1 effects as MYC-dependent.^16, 18, 47^ Although some studies reported MYC expression predicts JQ1 sensitivity, our results indicate other mechanisms are relevant; as JQ1 reduced tumour cell growth both in MYC-amplified (MCF-7 and HCC1806) and MYC-non-amplified cell lines (MDA-MB-231, Cal51 and SUM159). Our data provide further evidence for the context dependence of BETi.

Several studies show that BETi have broader MYC-independent effects.^19, 48, 49^ JQ1 impairs the recruitment of multiple TFs to their targets by physical disruption, for example, between the BRD4 and the N-terminal domain of the androgen receptor.^19, 21^ Thus, JQ1 acts by blocking BET protein ability to bind to chromatin, which in turn prevents TF recruitment, possibly including HIF. This is in agreement with our observation of reduced HIF binding to the *CA9* promoter region in response to JQ1. Other important hypoxia-regulated genes demonstrated a similar pattern of downregulation by JQ1 in this study. These include CXCR7 and LOX. CXCR7 is a G protein-coupled receptor upregulated in breast cancer associated with worst outcome that mediates angiogenesis and metastasis.^50^ LOX is also upregulated in breast cancer and confers a poor prognosis, where it enables angiogenesis^51^ and disrupts bone homeostasis providing a favourable environment for metastatic cells from hypoxic ER− breast cancer.^52^ Taken together, this led us to the original suggestion that HIF targets can be divided into BET-dependent and BET-independent.

The SWI/SNF chromatin remodelling complex was the first epigenetic factor demonstrated to regulate the response to hypoxia.^5, 53^ This complex makes DNA accessible to other factors, especially through its ATPase subunits BRM and BRG1.^54^ In breast cancer, BRG1 and BRM are overexpressed in most primary breast cancers and are needed for *in vivo* tumour formation and TNBC cell line proliferation.^55^ SWI/SNF can either directly regulate the expression of HIF-1α and HIF-2α or regulate the expression of hypoxia-responsive genes, including *CA9*.^5^ The *CA9* promoter nucleosome is BRG1-depedently remodelled in response to hypoxia.^5^ JQ1 does not bind to BRM or BRG1,^16^ and there is no current knowledge regarding interactions between BET proteins and SWI/SNF complex. While JQ1 prevents acetylated histones from being ‘read', SWI-SNF can promote deacetylation.^54^ Both interact with MYC.^16, 18, 47, 54^ Finally, just as CA9 was modulated by both factors, LDHA was not. Future studies should address to what extent the set of genes affected by these factors overlap. It might be the case that BET proteins and SWI/SNF complex interact at some level forming an enhanceosome and only some HIF targets are epigenetically regulated, rather than being BET- or SWI/SNF-dependent.

In conclusion, we showed that BETi impairs tumour response to hypoxia, targeting multiple pathways such as angiogenesis and pH control. Our findings alter the understanding of tumour response to hypoxia and identify a new avenue for epigenetic therapy to target the hypoxic tumour microenvironment. Furthermore, these results have a clear impact on the interpretation of the results from current clinical trials and future clinical use of drugs that inhibit the BET proteins in solid tumours.

## Materials and Methods

### Cell culture

Cells were maintained in DMEM+10% FBS at 5% CO_2_, 37 °C. Hypoxic incubations: 0.1%O_2_ in INVIVO2400 workstation (Baker Ruskinn, Sanford, ME, USA). Cell number was measured by Sulforhodamine B assay^56^ or CyQUANT (Molecular Probes, Waltham, MA, USA) following the manufacturer's instructions. Cell lines were purchased from ATCC (Manassas, VA, USA; MDA-MB-231, HCC1806, MCF-7), Creative Bioarray (Shirley, NY, USA) (CAL51) and Asterand (Royston, UK) (SUM159); these have stringent quality control for cell authenticity incorporating short tandem repeat profiling. Cells were regularly tested for mycoplasma. Stefen Knapp provided JQ1 (University of Oxford, UK) and Daniel Ebner provided I-BET151 and I-BET762 (University of Oxford, UK).

### Spheroid culture

Cells were seeded in round-bottomed plates (Corning, Corning, NY, USA) with Matrigel (BD Bioscience, Franklin Lakes, NJ, USA)-supplemented media, aggregated by centrifugation (2000 r.p.m./10 min). Treatment was daily renewed. Pictures were taken 3 days/week with an inverted microscope (EVOS xl Core, AMG, Waltham, MA, USA) (*n*=3).

### Gene expression array and ChIP-Seq data analysis

Illumina whole genome gene expression was performed (*n*=3 per group). Biotin-labelled aRNA was hybridized, according to the manufacturer's instruction (Illumina Inc., San Diego, CA, USA; #11286340), to high-density Illumina Human oligonucleotide arrays Human HT-12_V4_0_R1_15002873_B. Data are available at Array Express (https://www.ebi.ac.uk/arrayexpress/experiments/E-MTAB-4604/ E-MTAB-4604). Fluorescence emissions were detected using iScanner and data were extracted using BeadStudio v2011.1 Software (Illumina Inc) and imported to GeneSpring GX 12.1 (Agilent Technologies, Inc., Santa Clara, CA, USA); Illumina microarray data were pre-processed, normalized and differential expression analysed using R package LIMMA (v3.22.4). Significantly DEG were regarded as those with false discovery rate (Benjamini–Hochberg) corrected *P* value cutoff of <0.01. Analyses were performed using R (v3.1.2).

Functional analysis was carried out on DEG to identify statistically overrepresented ontologies using Database for Annotation, Visualization and Integrated Discovery (https://david.ncifcrf.gov/). DEG fulfilled the criteria: FC log2⩾1 and *P*<0.05.

The prognostic value of DEG was generated using The Cancer Genome Atlas (TCGA) (more information available at http://cancergenome.nih.gov/).

Genecodis^28, 29^ (http://genecodis.cnb.csic.es/) was used to investigate pathway alterations. Hypergeometric test was used (false discovery rate=0.05). The list of genes comprising these pathways were obtained from KEGG (http://www.genome.jp/kegg/), GO or the study by Buffa *et al.*^27^ (Supplementary Table S3). The Hypoxic Response (HyR) network was designed in STRING (http://string-db.org/) containing the selected pathways (Figure 1b).

Gene Set Enrichment Analysis (v2.1) was used to evaluate pathway enrichment^57^ (Supplementary Table S3). ViaComplex (v1.0)^58^ was used to generate representative landscape images of these results (Figure 1c and Supplementary Figure S1B).

Publicly available MCF-7 ChIP-Seq data (GEO dataset GSE55923) was analysed using MACS2.^59^ Sequence alignments were performed using Bowtie v1 (parameters: -v 2 -m 1 -3 1 -S 2) against human genome assembly hg19. Peaks were called using MACS2 (parameters: —bdg —nomodel —extsize 283.67 —gsize 2.7e9 —pvalue 1e-3). Peaks were annotated with gene names (UCSC Hg19 gene annotations version: September 2014) if they overlapped within 10 000 bp upstream (strand-specific) or within gene body. For comparisons, each BRD4 replicate was independently compared with the same MCF-7 Input control. Peaks of three MCF-7 BRD4 replicates were compared for pairwise correlation using bigWigCorrelate. Correlation results suggested only modest correlation (rho 0.4-0.55), therefore replicates were deemed unsuitable for pooling.

### Real-time PCR (qPCR)

qPCR was performed (*n*=3) as described previously.^35^ Primers sequences are available in Supplementary Table S4.

### Immunoblotting

Immunoblotting was performed as described previously (*n*=3)^35^ with primary antibodies listed in Supplementary Table S7. Bands were quantified using ImageJ.

### Xenograft studies

Mice were housed at BMS, University of Oxford, UK, and procedures were carried out under a Home Office licence (PPL30/2771). Female Crl:NU-Foxn1^nu^ mice (Charles River, Oxford, UK) (6–7-week-old) were injected orthotopically into the mammary fat pad with 2.5 × 10^6^ HCC1806 cells in 1:1 serum-free medium and Matrigel (BD Bioscience) (*n*=5 animals per group).

Tumour growth was measured with calipers by experienced technicians blinded to the experimental hypothesis and after three out of five animals reached 150 mm^3^, animals received JQ1 or vehicle (10% DMSO, 10% hydroxypropyl beta cyclodextrin) intraperitoneally at 50 mg/kg daily. Animals were randomly grouped at injection and one group was treated with JQ1 whilst the other was untreated (mean tumour sizes at the treatment starts were; shCTL, 120 mm^3^ and JQ1-treated, 137 mm^3^). When tumours reached 1.44 cm^3^, the mice were killed by cervical dislocation.

### Immunohistochemistry

Immunohistochemistry was carried out as previously described (*n*=3 untreated, *n*=5 JQ1-treated).^35^ Slides were submitted to antigen retrieval, and the primary antibodies listed in Supplementary Table S7. Slides were incubated with secondary antibody (Dako, Cambridgeshire, UK) and DAB (Dako) and counterstained with haematoxylin solution (Sigma-Aldrich Corp., St Louis, MO, USA). Secondary-only control staining was performed routinely. ImageJ colour deconvolution was used for quantification.^35^

### Gene silencing by RNA interference

Transfections of siRNA duplexes targeting BET proteins (BRD2–4 and BRDT) or a scramble control (ON-TARGETplus SMARTpool) were performed in Optimem (Invitrogen, Waltham, MA, USA), using Oligofectamine (Invitrogen) (*n*=3).

### ChIP assay

ChIP assay was performed for antibodies listed in Supplementary Table S7 using the EZ-ChIP Chromatin Immunoprecipitation Kit (#17-371, Millipore, Billerica, MA, USA) or ChIP-IT Express Enzymatic (Active Motif) according to the manufacturer's instructions. DNA isolated from ChIP was quantified by qPCR using CA9 or VEGF promoter primers (Supplementary Table S4) (*n*=3).

### Statistical analysis

Statistical analysis and graphs were performed using GraphPad Prism v6.0 (GraphPad, La Jolla, CA, USA). Results are plotted as mean values with standard deviation. Statistical tests and the number of repeats are described in the figure legends. Student's *t*-test was used for two sample analyses and normal distributions were assumed, otherwise the non-parametric Mann–Whitney test was used. Analysis of variance was used for >2 sample analyses. No samples or experimental repeats were excluded from analyses. For the *in vivo* experiment to detect a FC of 2 at alpha (*P* value)= 0.05, five animals in each group gave a 75% power of detection. No statistical methods were used for the samples size selection of other experiments.
