# Supplementary material for: The BET inhibitor JQ1 selectively impairs tumour response to hypoxia and downregulates CA9 and angiogenesis in triple negative breast cancer
Source: Oncogene. 2016 Jun 13;36(1):122–32. doi: 10.1038/onc.2016.184 (PMC5061082; doi:10.1038/onc.2016.184)
Supplement: Supplementary Figures Legends [file onc2016184x1.docx]

# Supplementary Figure Legends

**Fig.S1. JQ1 downregulates expression of several hypoxia-regulated genes, especially CA9.** (A) List of differentially expressed genes (DEG) under hypoxia for MCF-7 cells obtained from microarray. Columns at the right denote DEG under JQ1 treatment, either in normoxia (red blocks) or hypoxia (blue dots). (B) Many components of HyN are upregulated by hypoxia and downregulated by JQ1 treatment in MCF-7 cells. (C) Median logFC of genes modulated by JQ1. Bars represent CI: 5-95 and median. Non-parametric t-test: Mann-Whitney.

**Fig.S2. Genes consistently modulated by JQ1 in both cell lines tested that do not present prognostic value for TNBC patients.**

**Fig.S3. JQ1 reduces TNBC monolayer and spheroid growth.** (A) (+)-JQ1 reduces monolayer (2D) cell growth of TNBC cell lines Cal51 and SUM159 in normoxic and hypoxic conditions after 72h incubation, while (-)-JQ1 does not. (B) Densitometry of c-Myc immunocontent in MDA-MB-231 and HCC1806 cell lines with and without (+)-JQ1 treatment. (C) Spheroid growth curves for HCC1806 and Cal51 cell lines following JQ1 treatment, (+)-JQ1 reduces spheroid growth in the cell lines, while (-)-JQ1 does not. One-way ANOVA, n = 3, * p < 0.05, ** p < 0.01, *** p < 0.001.

**Fig.S4. JQ1 reduces CA9 expression in TNBC cell lines.** (A) Gene expression alterations due to JQ1 treatment in normoxia. CA9 is consistently downregulated by JQ1, without any effect on HIF. Cells were treated with JQ1 for 24h prior to RNA or protein extraction, then gene expression was assessed by RT-qPCR. Two-way ANOVA, n = 3, * p < 0.05, ** p < 0.01, *** p < 0.001.

**Fig.S5. I-BET-151 and I-BET-762 reduces CA9 expression in TNBC cell lines.** Other BET inhibitors induce similar gene expression modulation in TNBC cell lines HCC1806 (A) and MDA-MB-231 (B). Cells were treated with 1µM I-BET-151 or I-BET-762 for 24h prior to RNA extraction, and gene expression was assessed by RT-qPCR. Two-way ANOVA, n = 3, * p < 0.05, ** p < 0.01, *** p < 0.001.

**Fig.S6. Expression of BET.** Relative expression of BET proteins following siRNA knockdown (A) or incubation in normoxia and hypoxia (B). One-way ANOVA for (A) and student t-test for (B), n=3. (C) ChIP assay for VEGF promoter region with BRD4, H3K27 acetylation or H4 acetylation immunoprecipitation in HCC1806. T-Test, n=3. (D) ChIP assay for CA9 promoter region with BRD4, H3K27 acetylation or H4 acetylation immunoprecipitation in MDA-MB-231. T-Test, n=3, *p<0.05; **p<0.01. * p <0.05; ** p < 0.01; *** p < 0.001.
