# Supplementary material for: The BET inhibitor JQ1 selectively impairs tumour response to hypoxia and downregulates CA9 and angiogenesis in triple negative breast cancer
Source: Oncogene. 2016 Jun 13;36(1):122–32. doi: 10.1038/onc.2016.184 (PMC5061082; doi:10.1038/onc.2016.184)
Supplement: Supplementary Tables Legends [file onc2016184x2.docx]

***Supplementary table legends:***

**Table S1** - List of genes in each pathway that compose the Hypoxia Network (HyN)

**Table S2** - Gene Set Enrichment Analysis (GSEA) results for each condition and pathway in MD-MB-231 cells

**Table S3** - Gene Set Enrichment Analysis (GSEA) results for each condition and pathway in MCF-7 cells

**Table S4** - Sequence of primers used for qPCR and ChIP

**Table S5** - List of genes simultaneously downregulated by JQ1 in hypoxia and bound in normoxia to BRD4 by ChIP analysis in MCF7 cells

**Table S6** - List of genes the are bound to BRD4 in normoxia and to HIF1α or HIF2α in hypoxia in published data sets with MCF7 cells

**Table S7** - List of antibodies used for western blot, immunohistochemistry or ChIP
