# Supplementary material for: The BET inhibitor JQ1 selectively impairs tumour response to hypoxia and downregulates CA9 and angiogenesis in triple negative breast cancer
Source: Oncogene. 2016 Jun 13;36(1):122–32. doi: 10.1038/onc.2016.184 (PMC5061082; doi:10.1038/onc.2016.184)
Supplement: Supplementary Figure [file onc2016184x10.ppt]

## Slide 1
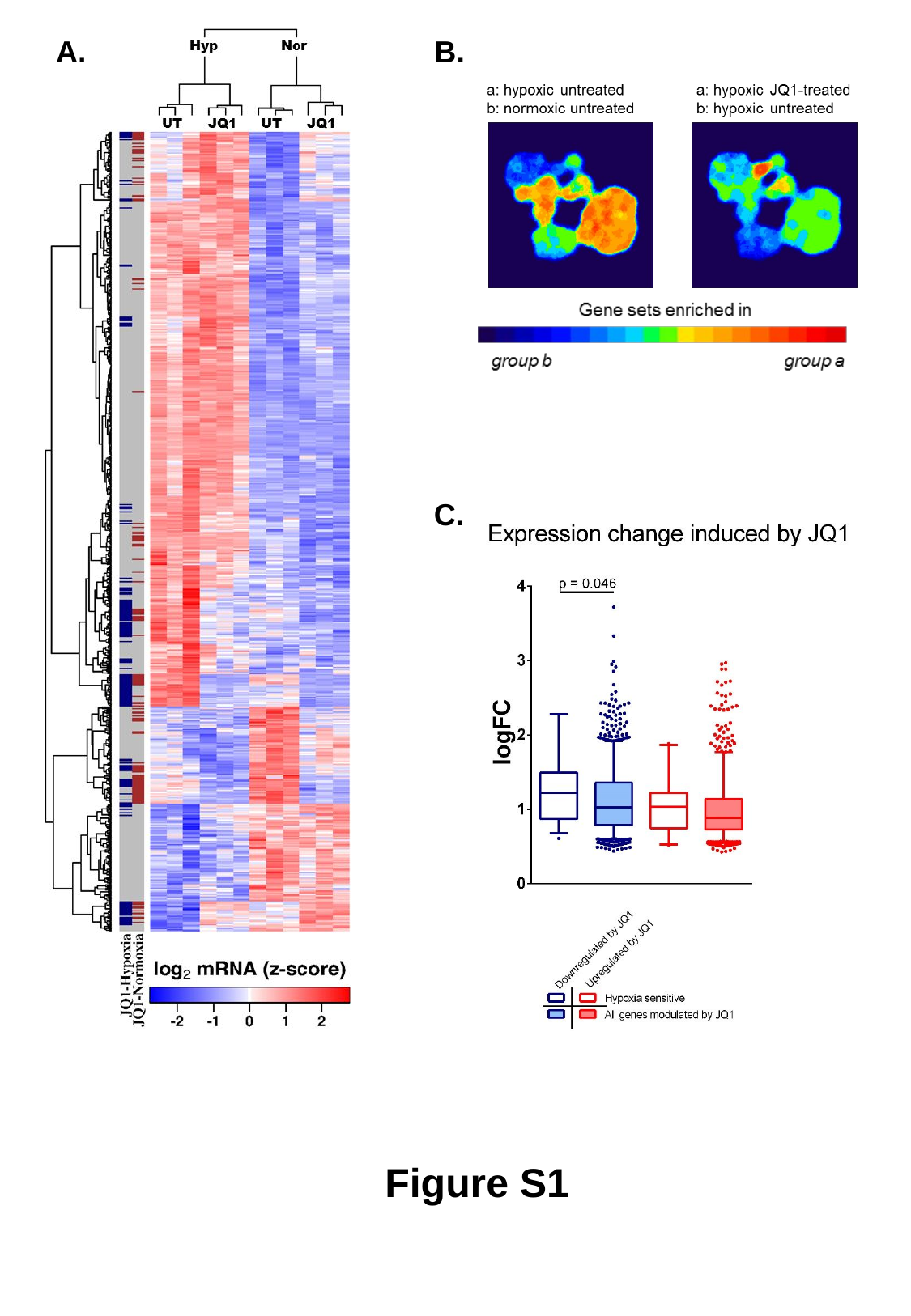

A.
B.
C.
Figure S1

## Slide 2
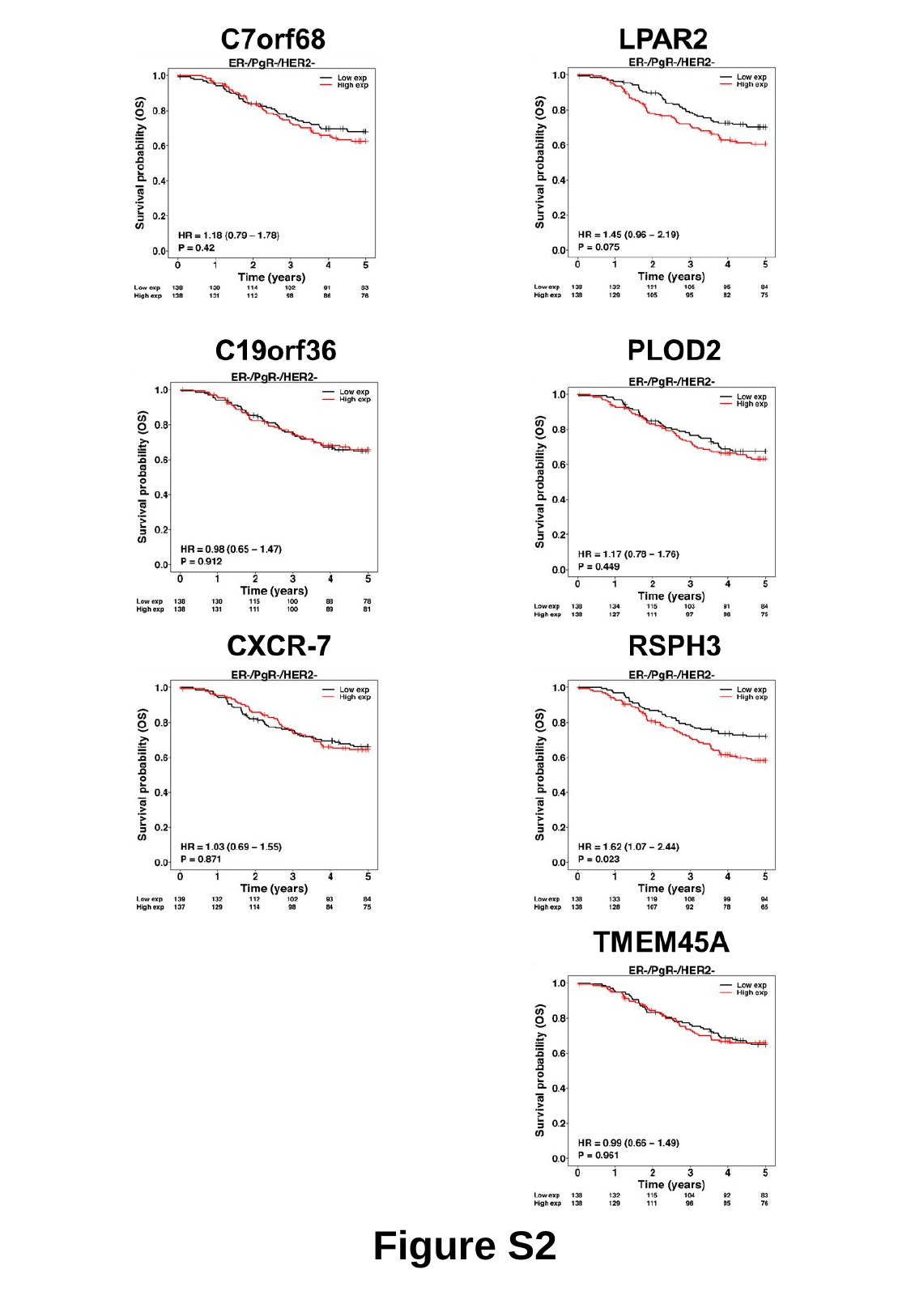

Figure S2

## Slide 3
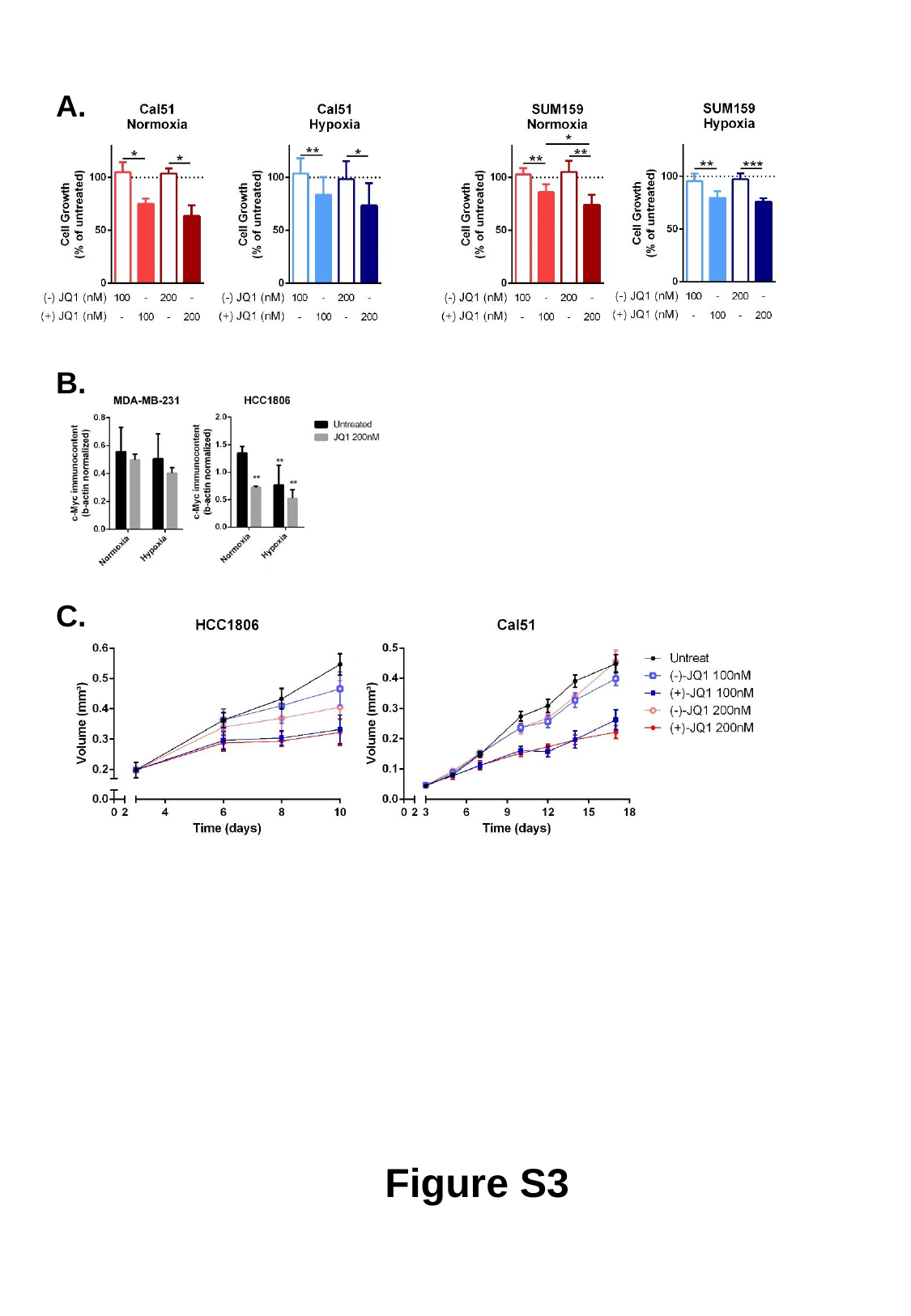

A.
B.
C.
Figure S3

## Slide 4
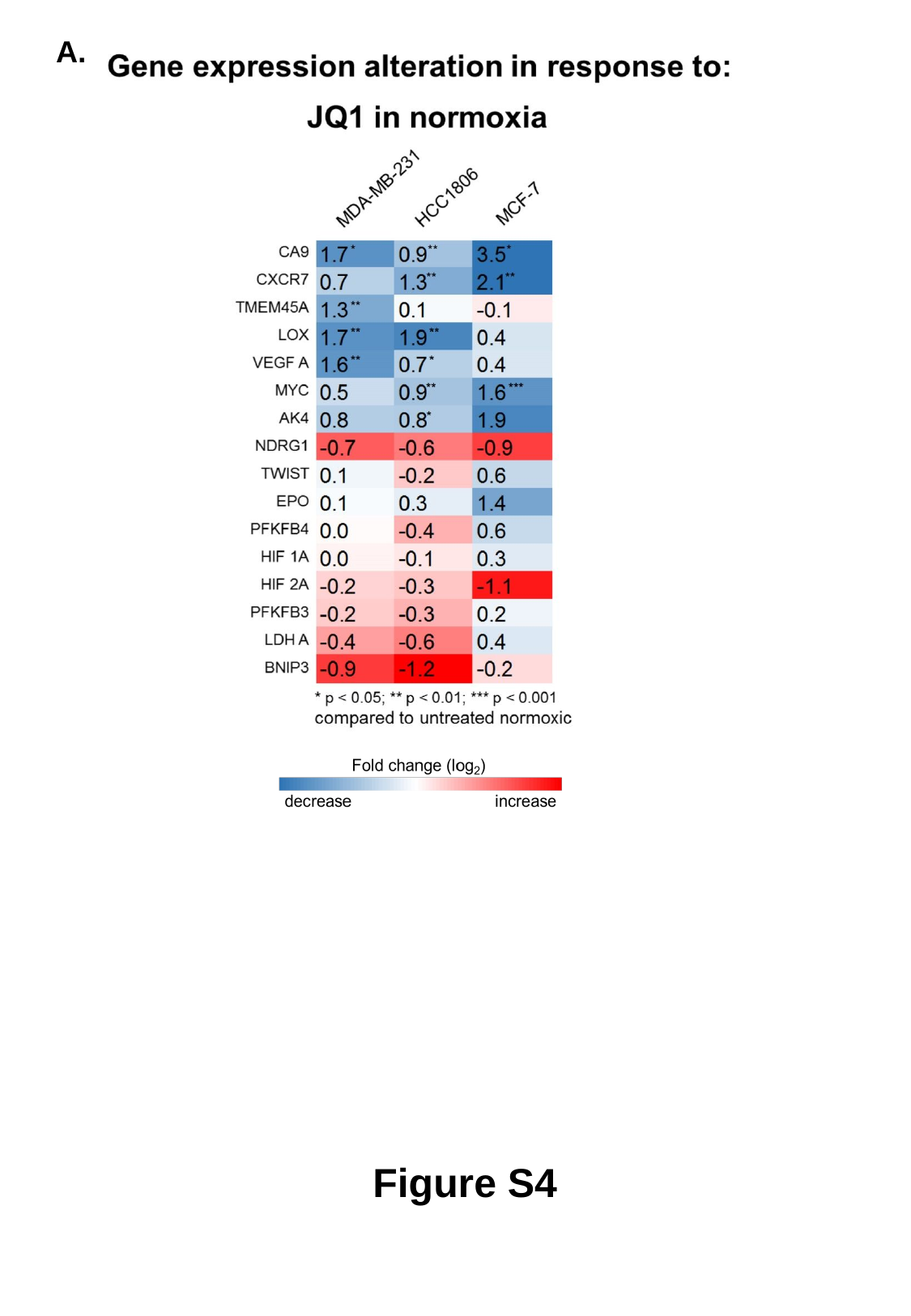

A.
Figure S4

## Slide 5
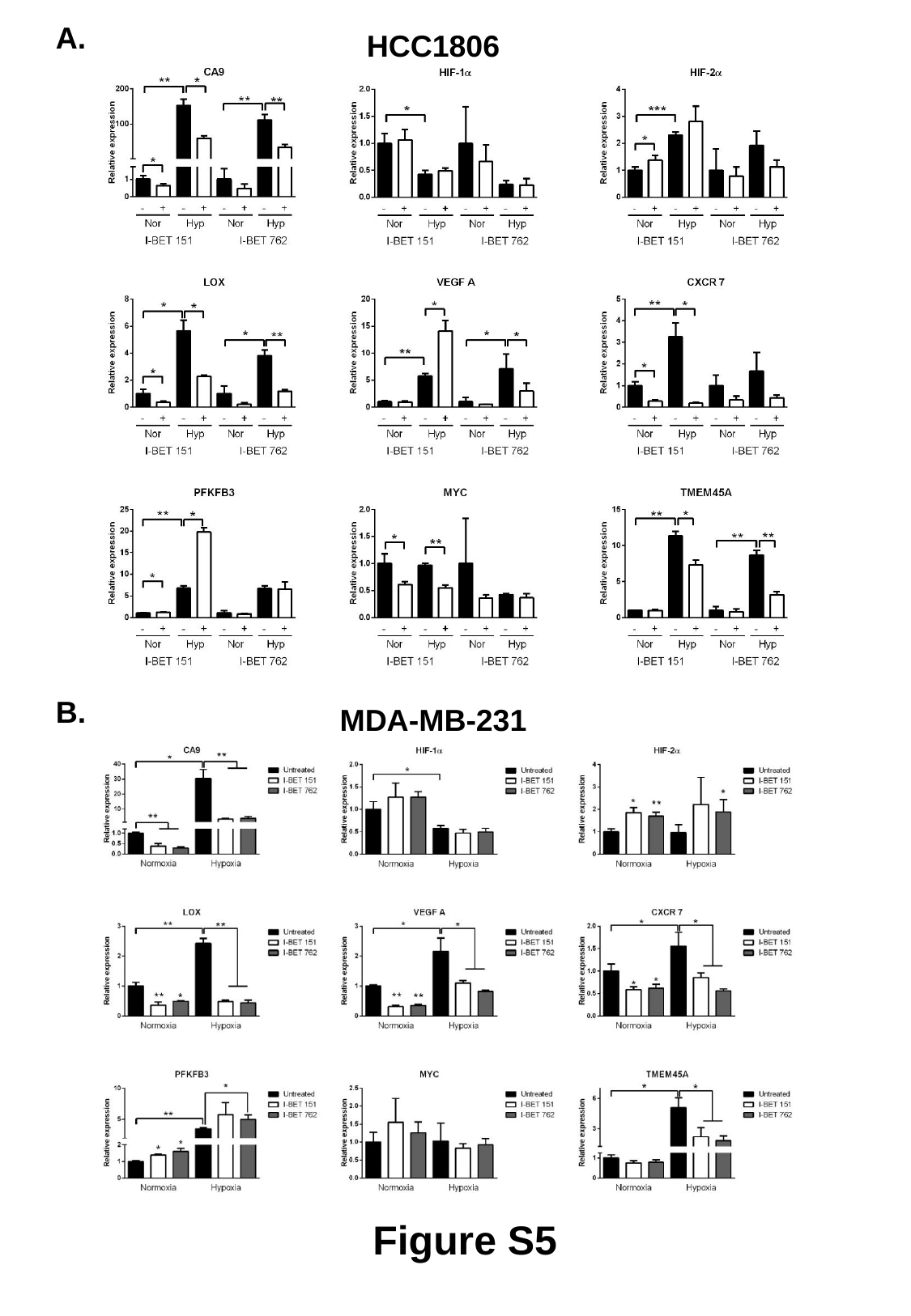

A.
HCC1806
B.
MDA-MB-231
Figure S5

## Slide 6
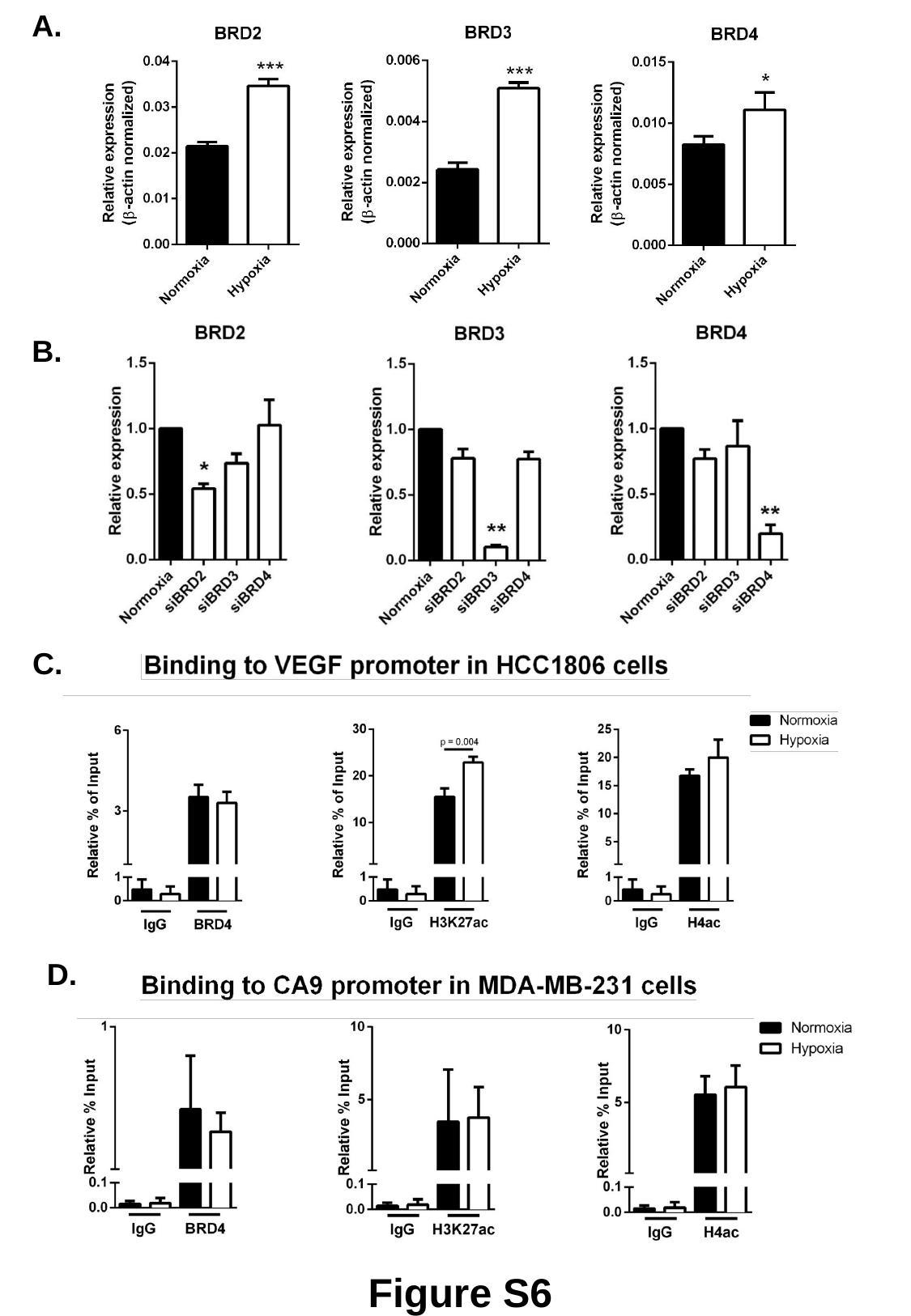

A.
B.
C.
D.
Figure S6
